# Children with Psychogenic Nonepileptic Seizures

**DOI:** 10.15844/pedneurbriefs-33-4

**Published:** 2019-12-31

**Authors:** Aisha Gillan, Safiullah Shareef

**Affiliations:** 1 University of North Texas Health Science Center, Fort Worth, TX; 2 Texas Child Neurology, Plano, TX

**Keywords:** Psychogenic Nonepileptic Seizures, Epilepsy, Children, PNES

## Abstract

A large multicenter retrospective cohort study was conducted by researchers from the Pediatric Health Information System hospital network to determine differences in demographics, clinical characteristics, testing, treatment, and healthcare use between children aged 8–20 years with epilepsy (*n* = 13,241) and those with psychogenic nonepileptic seizures (PNES) secondary to conversion disorder (*n* = 399).

A large multicenter retrospective cohort study was conducted by researchers from the Pediatric Health Information System hospital network to determine differences in demographics, clinical characteristics, testing, treatment, and healthcare use between children aged 8–20 years with epilepsy (*n* = 13,241) and those with psychogenic nonepileptic seizures (PNES) secondary to conversion disorder (*n* = 399). Inpatient diagnosis of PNES was defined as having two simultaneous diagnosis codes—one for conversion disorder (300.11) and the other for “other convulsions” (780.39)—with one of them being the principal diagnosis. [1]

COMMENTARY. Conversion disorder remains a chronic and complex psychiatric process [2], and PNES specifically constitute one of three most common diagnoses for patients presenting with a transient loss of consciousness [3]. No single etiology has been identified for PNES as they are understood to be multifactorial, biopsychosocial disorders that are triggered when underlying genetics, childhood experiences, and temperament traits are combined with precipitating and perpetuating stressors. The current hypothesis for the pathophysiology of PNES involves a dysfunction of cerebral arousal and inhibitory networks [4]. Scant data exist regarding the differences in clinical characteristics and the comorbidities associated with PNES.

This study highlights the importance of screening PNES patients for mental health disorders by taking into consideration the high percentage of patients that have comorbid mental health conditions in PNES compared to patients with epilepsy. In addition, it proposes a higher index of suspicion for PNES in patients with mental health disorders or pain disorders. A unique finding of this study was that fewer pediatric PNES patients were hospitalized during the summer compared to those with epilepsy, further identifying a link between PNES and seasonal stressors.

Although this study showed a link between psychiatric diagnosis and PNES, the rates of psychiatric diagnoses were lower in both cohorts as compared to other studies. This may be due to differences in the study design, as this study did not include any outpatient diagnoses or diagnoses made after patient discharge.

One significant limitation of this study is the exclusion of patients with PNES who also had comorbid epilepsy (PNES+epilepsy). All records with diagnosis codes that may predispose patients to epilepsy were excluded, as were records with a psychiatrist as the admitting provider. Previous studies have found that PNES are associated with epilepsy, along with other neurological disorders. This study focused only on patients with PNES who did not have epilepsy (PNES-w/o-epilepsy). In prior published case series of patients with PNES, it has been demonstrated that the prevalence of epilepsy is increased in patients with PNES [3]. However, the most robust studies indicate that no more than 10% of adults with PNES have concurrent epilepsy. What is unclear is whether PNES+epilepsy has the same features as PNES-w/o-epilepsy. Therefore, readers should be careful not to generalize the findings of this study to all cases of PNES, especially PNES+epilepsy.

Based on the findings, this study recommends implementing longitudinal mental healthcare with a diagnosis of PNES. Furthermore, the findings highlight the importance of conducting further research on school-based stress reduction for children in whom school attendance is a PNES trigger. The study also recommends that hospitals should benefit from adjusting mental health provider staffing to account for seasonal variations.

## Disclosures

The author(s) have declared that no competing interests exist.
